# Prognostic value of pretherapeutic FDG PET/CT in non-small cell lung cancer with pulmonary lymphangitic carcinomatosis

**DOI:** 10.1038/s41598-022-24875-2

**Published:** 2023-01-07

**Authors:** Yong-Jin Park, Yunjoo Im, O. Jung Kwon, Joungho Han, Myung-Ju Ahn, Jhingook Kim, Sang-Won Um, Joon Young Choi

**Affiliations:** 1 Department of Nuclear Medicine, Samsung Medical Center, Sungkyunkwan University School of Medicine, 81, Irwon-Ro, Gangnam-Gu, Seoul, Republic of Korea; 2 Department of Nuclear Medicine, Ajou University School of Medicine, Suwon, Republic of Korea; 3 Division of Pulmonary and Critical Care Medicine, Department of Medicine, Samsung Medical Center, Sungkyunkwan University School of Medicine, 81, Irwon-Ro, Gangnam-Gu, Seoul, Republic of Korea; 4 Department of Pathology, Samsung Medical Center, Sungkyunkwan University School of Medicine, Seoul, Republic of Korea; 5 Division of Hematology-Oncology, Department of Medicine, Samsung Medical Center, Sungkyunkwan University School of Medicine, Seoul, Republic of Korea; 6 Department of Thoracic and Cardiovascular Surgery, Samsung Medical Center, Sungkyunkwan University School of Medicine, Seoul, Republic of Korea

**Keywords:** Lung cancer, Oncology

## Abstract

Pulmonary lymphangitic carcinomatosis (PLC) is associated with a poor prognosis in patients with non-small cell lung cancer (NSCLC). We sought to determine prognostic value of pretherapeutic fluorine-18-fluorodeoxyglucose (FDG) positron emission tomography (PET)/computed tomography (CT) in NSCLC with radiologically diagnosed PLC. We retrospectively reviewed 50 NSCLC patients with radiologically diagnosed PLC. Among eight clinical variables and five imaging parameters, metabolic PLC burden, which represents the overall tumor burden of PLC, and cPLC, which represents the location and extent of PLC in a three-grade system, were used. In multivariate analyses for progression-free survival, metabolic PLC burden (P = 0.0181), cPLC (P = 0.0401), and clinical stage (P = 0.0284) were identified as independent prognostic factors. High metabolic PLC burden had a worse prognosis, and the prognosis of cPLC3 was significantly worse than that of cPLC1 or cPLC2. In univariate analyses for overall survival, only age (P = 0.0073) was identified a prognostic factor. In conclusion, FDG PET/CT parameters were identified as independent prognostic factors in NSCLC with radiologically diagnosed PLC. Furthermore, a combination of anatomical and metabolic information about PLC obtained using FDG PET/CT provides insight into the overall tumor burden of PLC and is useful in predicting prognosis.

## Introduction

Lung cancer has a five-year survival rate of only 15% and is known to be the leading cause of cancer-related deaths worldwide^1^. It accounts for 1.5–1.7 million deaths annually^2^. About 85–90% of lung cancers are of non-small cell lung cancer (NSCLC), and most patients with NSCLC are in advanced stages^2^. Pulmonary lymphangitic carcinomatosis (PLC), defined as the dissemination of tumor into the lymphatics in the lung^3^, was first described by Troisier in 1873^4^. PLC may be localized to small areas or may be widely distributed over large areas, and it is frequently associated with advanced NSCLC^3^. PLC is mainly an advanced stage of cancer^5^, and therefore it has been realized as an indicator of poor survival^6^. PLC is related to the movement of tumor emboli into adjacent vessels or lymphatics, and tumor emboli explain the poor prognosis in advanced NSCLC patients with PLC^3^.

Although development of PLC is known to be generally associated with a poor prognosis^7^, a descriptor for PLC was not included in either the 7th or 8th editions of the American Joint Committee on Cancer (AJCC) tumor-node-metastasis (TNM) staging system^8^. For that reason, editors of International Association for the Study of Lung Cancer (IASLC) Staging Project proposed to categorize PLC as an independent descriptor, cLy, with a four-grade system based on PLC extents^8^. However, the prognostic implications of PLC were difficult to confirm due to small sample size and lack of significant difference in one-year prognosis of different classifications of PLC^7,8^. In addition, few cLy data were available in the IASLC database, which mainly included patients who underwent surgery^2^. Therefore, further studies on the prognosis of PLC have been continuously needed.

To date, few previous studies have been published on the prognostic value of PLC in patients with NSCLC. In a previous prognosis study associated with extents of PLC, Im et al. reported that patients with PLC is confined to the lobe of the primary tumor, had a better overall survival than those with PLC is in other ipsilateral lobes or the contralateral lung, or intrapulmonary metastases in patients with NSCLC^2^. In another previous study using fluorine-18-fluorodeoxyglucose (FDG) positron emission tomography (PET)/computed tomography (CT) in patients with advanced NSCLC, authors suggested that total lesion glycolysis of primary tumor was a good predictive factor in the PLC group. To date, however, no previous prognostic studies using FDG PET parameters of PLC have been published in NSCLC. Therefore, combining the extents of PLC and FDG PET parameters of PLC was thought to suggest overall PLC burden, and it might also have the potential as a new prognostic factor in NSCLC. Furthermore, in previous studies, extents of PLC were divided into focal and diffuse, and distribution of PLC within one lung lobe was called focal PLC^3,9^. Using the concept of focal PLC, we thought it could be useful to change the previous four-grade system (cLy), which represents the location and extent of PLC, to a simpler grade system.

In the present study, we sought to determine prognostic value of pretherapeutic FDG PET/CT in NSCLC with radiologically diagnosed PLC. Especially, it was confirmed whether PLC PET parameters representing PLC burden was possible as independent prognostic factors. In addition, a simpler three-grade system (cPLC) was newly used instead of the known four-grade system (cLy), and it was also confirmed that there is a possibility of an independent prognostic factor. Through these results, the clinical usefulness of FDG PET/CT in NSCLC patients with radiologically diagnosed PLC was investigated.

## Results

### Patient characteristics

A total of 50 study patients were enrolled and the patient characteristics were summarized (Table 1). The patients consisted of 35 men (70%) and 15 women (30%), and the median age of patients was 61.50 years (range of 37–77 years). During the 5-year follow-up period, 14 (28%) were found to have disease progression and 17 (34%) died. Median progression-free survival (PFS) and overall survival (OS) were 11.00 and 31.00 months, respectively. In the initial staging using the eighth edition of the AJCC TNM staging system, clinical stage II, III, and IV were one (2%), 43 (86%), and six (12%), respectively. Of 44 patients who underwent chemotherapy, patients underwent chemotherapy with/without target therapy or immunotherapy, epidermal growth factor receptor (EGFR)-targeted therapy, and EGFR-targeted therapy with immunotherapy were 40, 3, and 1 respectively. Of the total patients, 29 (58%) and 21 (42%) patients received curative-intent treatment and palliative treatment. Median primary tumor size and maximum standardized uptake value (SUV_max_) were 43.50 mm (range of 12–69 mm) and 11.15 (range of 3.47–32.00), respectively. Primary tumor SUV_max_ measured by two physicians showed consistent results (kappa coefficient = 1). Among the cPLC groups, cPLC1 (n = 37, 74%) accounted for the highest proportion, and the remaining cPLC2 and cPLC3 were identified as seven (14%) and six (12%). Regarding number of lobes with PLC, 37 (74%) had one lobe with PLC, and six (12%) had two lobes with PLC. There were seven (14%) with three or more lobes with PLC. It was confirmed that location and number of lobes containing PLC on high-resolution computed tomography (HRCT) and CT of FDG PET/CT was the same. Median PLC SUV_max_ and metabolic PLC burden were 2.03 (range of 1.05–8.39) and 2.16 (range of 1.05–21.00), and the median metabolic PLC burden had a slightly higher value than the median PLC SUV_max_. PLC SUV_max_ measured by two physicians showed very good strength of agreement (kappa coefficient = 0.98666).

**Table 1:** Baseline characteristics of 50 study patients.

	Number of patients (%)	Median (range)
Age (years)		61.50 (37–77)
**Sex**		
Male/female	35 (70)/15 (30)	
Progression	14 (28)	
Death	17 (34)	
PFS (months)		11.00 (1–60)
OS (months)		31.00 (1–60)
**Smoking**		
Never smoked	14 (28)	
Ex-smoker	21 (42)	
Currently smoking	15 (30)	
Pack year		21.50 (0–100)
**Clinical stage**		
IIB	1 (2)	
IIIA	5 (10)	
IIIB	25 (50)	
IIIC	13 (26)	
IVA	6 (12)	
**Primary tumor histology**		
Adenocarcinoma	31 (62)	
Squamous cell carcinoma	17 (34)	
Other NSCLCs	2 (4)	
**ALK mutation**		
Positive	4 (8)	
Negative	26 (52)	
Not available	20 (40)	
**EGFR mutation**		
Positive	10 (20)	
Negative	17 (34)	
Not available	23 (46)	
**Treatment modality**		
Curative-intent treatment	29 (58)	
Palliative treatment	21 (42)	
Primary tumor size (mm)		43.50 (12–69)
**cPLC**		
cPLC1	37 (74)	
cPLC2	7 (14)	
cPLC3	6 (12)	
**Number of lobes with PLC**		
1	37 (74)	
2	6 (12)	
3	2 (4)	
4	2 (4)	
5	3 (6)	
Primary tumor SUV_max_		11.15 (3.47–32.00)
PLC SUV_max_		2.03 (1.05–8.39)
Metabolic PLC burden		2.16 (1.05–21.00)

*PFS* progression-free survival, *OS* overall survival, *NSCLC* non-small cell lung cancer, *ALK* anaplastic lymphoma kinase, *EGFR* epidermal growth factor receptor, *PLC* pulmonary lymphangitic carcinomatosis, *SUV*_*max*_ maximum standardized uptake value.

### Progression-free survival

**Figure 1:**
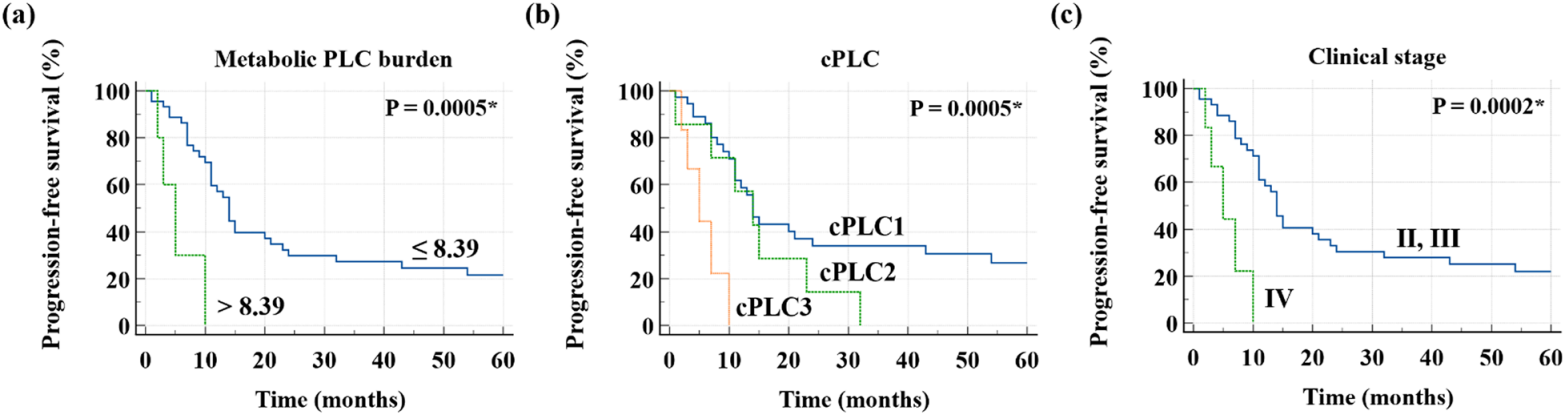
Kaplan–Meier curves and log-rank test of PFS with respect to (**a**) metabolic PLC burden, (**b**) cPLC, and (**c**) clinical stage. *PFS* progression-free survival, *PLC* pulmonary lymphangitic carcinomatosis. *P < 0.05.

**Figure 2:**
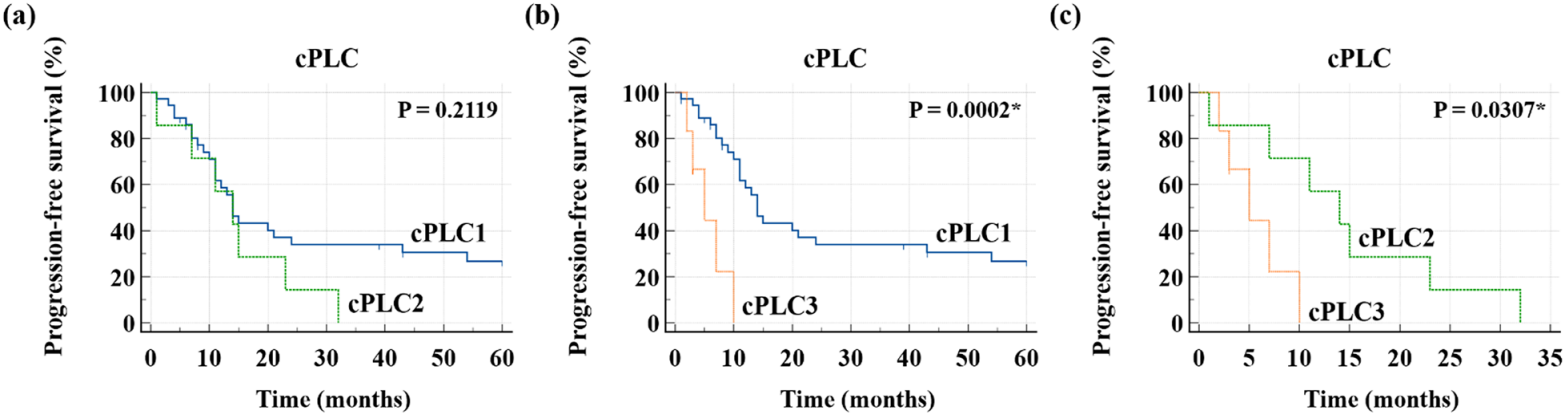
Comparison of Kaplan–Meier curves and log-rank test of PFS between (**a**) cPLC1 and cPLC2, (**b**) cPLC1 and cPLC3, and (**c**) cPLC2 and cPLC3. *PFS* progression-free survival, *PLC* pulmonary lymphangitic carcinomatosis. *P < 0.05.

In univariate analyses for PFS using eight clinical variables and five imaging parameters, P values of clinical stage, treatment modality, cPLC, primary tumor SUV_max_, and metabolic PLC burden were less than 0.05 (Table 2). In the prognosis analysis for PFS, optimal cutoff values of PLC SUV_max_, primary tumor SUV_max_, metabolic PLC burden, and age were determined to be 1.59, 6.86, 8.39, and 69, respectively. In univariate analyses for PFS, it was confirmed that there was no significant difference between two groups of PLC SUV_max_ (P = 0.3467). Variance inflation factors (VIFs) were calculated to detect multicollinearity of the five selected variables. Among the five variables, it was confirmed that the VIFs of metabolic PLC burden (VIF = 5.670), cPLC (VIF = 6.375), and clinical stage (VIF = 9.363) were higher than 3.3. To control the confounding effects, three models (Model 1, Model 2, and Model 3) containing each of the variables with a VIF greater than 3.3 were created, and the three generated models included the remaining three variables with a VIF less than 3.3. It was confirmed that VIFs of all variables of the three models were less than 3.3 (Supplementary Table S1). Finally, Model 1 was composed of metabolic PLC burden, primary tumor SUV_max_, and treatment modality, and Model 2 consisted of cPLC, primary tumor SUV_max_, and treatment modality. In addition, Model 3 was composed of clinical stage, primary tumor SUV_max_, and treatment modality. In multivariate analyses for PFS in the three models, P values of metabolic PLC burden (Model 1, P value = 0.0181, hazard ratio [HR] = 4.5373, 95% confidence interval [CI] 1.2949–15.8986), cPLC (Model 2, cPLC1 vs. cPLC3, P value = 0.0401, HR = 4.0577, 95% CI 1.0653–15.4553), and clinical stage (Model 3, P value = 0.0284, HR = 3.6535, 95% CI 1.1472–11.6354) were less than 0.05 (Table 3, Fig. 1). Therefore, metabolic PLC burden, cPLC, and clinical stage were independent prognostic factors for predicting PFS in NSCLC with PLC. In Kaplan–Meier curves with log-rank test of PFS, there was no significant difference in prognosis between cPLC1 and cPLC2 (P = 0.2119, HR = 1.9212, 95% CI 0.6892–5.3554, but there was a significant difference in prognosis between cPLC1 and cPLC3 (P = 0.0002, HR = 37.2853, 95% CI 5.5176–251.9561), and between cPLC2 and cPLC3 (P = 0.0307, HR = 6.0995, 95% CI 1.1836–31.4317) (Fig. 2).

**Table 2:** Univariate analysis for PFS.

	HR [95% CI]	P value
Age (> 69 years vs. ≤ 69 years)	0.2848 [0.0682–1.1890]	0.0850
Sex (male vs. female)	0.9007 [0.4494–1.8053]	0.7681
**Smoking**		0.6177
Never smoked	1	
Ex-smoker	0.8419 [0.3836–1.8476]	0.6678
Currently smoking	1.2648 [0.5557–2.8783]	0.5756
Clinical stage (IV vs. II, III)	5.8445 [2.002–17.0623]	0.0012*
Primary tumor histology (adenocarcinoma vs. squamous cell carcinoma)	1.2758 [0.5963–2.4285]	0.6052
ALK mutation (positive vs. negative)	1.2758 [0.3732–4.3613]	0.6977
EGFR mutation (positive vs. negative)	0.7274 [0.3043–1.7387]	0.4741
Treatment modality (palliative treatment vs. curative-intent treatment)	2.3702 [1.2082–4.6497]	0.0121*
**cPLC**		0.0099*
cPLC1	1	
cPLC2	1.6861 [0.7180–3.9594]	0.2304
cPLC3	6.4831 [2.1763–19.3128]	0.0008*
**Number of lobes with PLC**		0.0624
1	1	
2	1.6131 [0.6521–3.9922]	0.3007
3	3.7203 [0.8383–16.5091]	0.0840
4	8.3205 [1.7685–39.1452]	0.0073*
5	4.6304 [1.0081–21.2689]	0.0488*
Primary tumor SUV_max_ (> 6.86 vs. ≤ 6.86)	2.6064 [1.1693–5.8098]	0.0192*
PLC SUV_max_ (> 1.59 vs. ≤ 1.59)	1.4901 [0.6493–3.4198]	0.3467
Metabolic PLC burden (> 8.39 vs. ≤ 8.39)	5.9849 [1.8841–19.0110]	0.0024*

*PFS* progression-free survival, *HR* hazard ratio, *CI* confidence interval, *ALK* anaplastic lymphoma kinase, *EGFR* epidermal growth factor receptor, *PLC* pulmonary lymphangitic carcinomatosis, *SUV*_*max*_ maximum standardized uptake value.

*P < 0.05.

**Table 3:** Multivariate analysis for PFS.

	Model 1		Model 2		Model 3	
	HR [95% CI]	P value	HR [95% CI]	P value	HR [95% CI]	P value
Treatment modality (palliative treatment vs. curative-intent treatment)	1.6113 [0.7358–3.5281]	0.2329	1.4585 [0.4800–4.4317]	0.5056	1.6549 [0.7565–3.6202]	0.2072
Primary tumor SUV_max_ (> 6.86 vs. ≤ 6.86)	2.0866 [0.8662–5.0267]	0.1011	1.8196 [0.7158–4.6255]	0.2085	1.7323 [0.7158–4.1927]	0.2231
Metabolic PLC burden (> 8.39 vs. ≤ 8.39)	4.5373 [1.2949–15.8986]	0.0181*				
**cPLC**						
cPLC1			1			
cPLC2			1.2217 [0.3613–4.1318]	0.7473		
cPLC3			4.0577 [1.0653–15.4553]	0.0401*		
Clinical stage (IV vs. II, III)					3.6535 [1.1472–11.6354]	0.0284*

*PFS* progression-free survival, *HR* hazard ratio, *CI* confidence interval, *SUV*_*max*_ maximum standardized uptake value, *PLC* pulmonary lymphangitic carcinomatosis.

*P < 0.05.

### Overall survival

**Figure 3:**
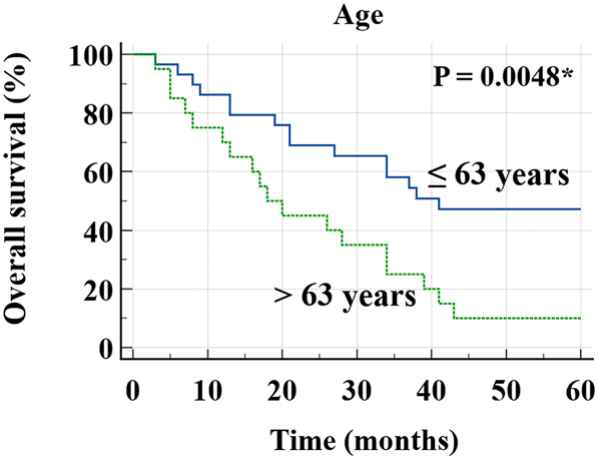
Kaplan–Meier curves and log-rank test of OS with respect to age. *OS* overall survival. *P < 0.05.

In univariate analyses for OS using eight clinical variables and five imaging parameters, P value of age was less than 0.05 (P value = 0.0073, HR = 2.5789, 95% CI 1.2910–5.1516) (Table 4). Multivariate analysis was not performed because only age was identified as a prognostic factor in univariate analyses for OS. Furthermore, imaging or PET parameters including PLC SUV_max_, primary tumor SUV_max_, cPLC, and metabolic PLC burden were not identified as prognostic factors in univariate analyses for OS. In the prognosis analysis for OS, optimal cutoff values of PLC SUV_max_, primary tumor SUV_max_, metabolic PLC burden, and age were determined to be 1.95, 15.79, 3.42, and 63, respectively. In Kaplan–Meier curves with log-rank test of age (Fig. 3), older patients (age > 63 years) showed significantly worse prognosis for OS than younger patients (age ≤ 63 years) (P = 0.0048, HR = 2.9313, 95% CI 1.3883–6.1891).

**Table 4:** Univariate analysis for OS.

	HR [95% CI]	P value
Age (> 63 years vs. ≤ 63 years)	2.5789 [1.2910–5.1516]	0.0073*
Sex (male vs. female)	0.6942 [0.3222–1.4958]	0.3392
**Smoking**		0.0772
Never smoked	1	
Ex-smoker	1.4072 [0.5741–3.4494]	0.4552
Currently smoking	2.6843 [1.1056–6.5169]	0.0291*
Clinical stage (IV vs. II, III)	1.7328 [0.6658–4.5098]	0.2600
Primary tumor histology (adenocarcinoma vs. squamous cell carcinoma)	1.6909 [0.8126–3.5183]	0.1600
ALK mutation (positive vs. negative)	4.8617 [0.6500–36.3652]	0.1235
EGFR mutation (positive vs. negative)	0.8086 [0.3222–2.0290]	0.6508
Treatment modality (palliative treatment vs. curative-intent treatment)	1.6913 [0.8528–3.3543]	0.1357
**cPLC**		0.3027
cPLC1	1	
cPLC2	1.7340 [0.7005–4.2921]	0.2339
cPLC3	1.9115 [0.7197–5.0768]	0.1936
**Number of lobes with PLC**		0.3466
1	1	
2	2.4324 [0.9739–6.0751]	0.0570
3	0.8333 [0.1122–6.1893]	0.8585
4	3.0652 [0.6935–13.5475]	0.1396
5	1.1784 [0.2766–5.0197]	0.8243
Primary tumor SUV_max_ (> 15.79 vs. ≤ 15.79)	1.8573 [0.6495–5.3110]	0.2487
PLC SUV_max_ (> 1.95 vs. ≤ 1.95)	1.5690 [0.7713–3.1919]	0.2138
Metabolic PLC burden (> 3.42 vs. ≤ 3.42)	1.6736 [0.8303–3.3738]	0.1499

*OS* overall survival, *HR* hazard ratio, *CI* confidence interval, *ALK* anaplastic lymphoma kinase, *EGFR* epidermal growth factor receptor, *PLC* pulmonary lymphangitic carcinomatosis, *SUV*_*max*_ maximum standardized uptake value.

*P < 0.05.

## Discussion

In the present study, we confirmed that metabolic PLC burden is an independent prognostic factor for PFS. This variable was developed as a concept in which the number of lobes containing PLC using CT was multiplied by PLC SUV_max_ using PET, and is thus based on anatomic and metabolic information of PLC. Therefore, the metabolic PLC burden could be used as one of the variables representing the tumor burden of the entire PLC. The newly used cPLC in this study was also identified as an independent prognostic factor for PFS. In the three-grade system of cPLC, the prognosis of patients with cPLC3 was significantly worse than that those with cPLC1 or cPLC2. Although the descriptor of PLC has not yet been included in the 7th and 8th editions of AJCC TNM staging system^8^, the results of this study are believed to be evidences for including the optional descriptor of PLC in the staging system. In addition, clinical stage (IV vs. II, III) was also identified as independent prognostic factors for PFS. Age (> 63 years vs. ≤ 63 years) was identified a prognostic factor for OS.

In this study, metabolic PLC burden, which was used as a variable representing the overall tumor burden of PLC, was identified as a significant predictor of five-year PFS. In order to represent the extent of the PLC, there are methods of representing the extent of the PLC based on the lobe in which the primary tumor is located^2,6,8,10^, such as cLy and cPLC, and the extent or distribution of the PLC may be represented by the number of lobes including the PLC. Im et al. found that patients with cLy1/2 had better overall survival than those with cLy3/4 or intrapulmonary metastases, and patients with cLy4 had a worse overall survival than those with intrapulmonary metastases^2^. Moreover, five-year OS rates of patients with cLy3 were no significantly different than those with intrapulmonary metastases^2^. Therefore, it was suggested that extent and location of PLCs are closely related to prognosis in patients with NSCLC. In the present study, the extent or distribution of PLC was shown using the number of lobes containing PLC or cPLC instead of cLy, and tumor burden of the entire PLC was expressed by multiplying the number of lobes containing PLC by PLC SUV_max_. FDG PET has been used as a sensitive tool to reflect increased tumor metabolism^11^. In previous studies, FDG uptake in PLC was significantly higher than that of normal lung^9,11^, and there is diffuse increased FDG uptake in the lung corresponding to the CT findings of PLC^11^. Furthermore, metabolic parameters of tumor and peritumoral areas and their respective ratios to background were significantly higher in patients with PLC than in those without PLC^4^, and sensitivity and specificity of FDG PET for PLC were 86% and 100%, respectively^9^. Therefore, it can be thought that the metabolic PLC burden obtained by multiplying the number of lobes containing PLC, which means extent of PLC, by PLC SUV_max_, which means tumor metabolism of PLC, means tumor burden for the entire PLC. In the present study, it was confirmed that patients with metabolic PLC burden greater than 8.39 had a worse prognosis for PFS than patients with metabolic PLC burden less than 8.39. In addition, since it has been confirmed that the HR of metabolic PLC burden (HR = 4.5373) using FDG PET/CT is greater than that of cPLC (HR between cPLC1 and cPLC3 = 4.0577) known through only CT, FDG PET with CT may be considered useful imaging modality in evaluating the overall tumor burden of PLC. Therefore, metabolic PLC burden is considered to be available as an independent prognostic factor for PFS in NSCLC with PLC, and FDG PET/CT is useful in evaluating the overall tumor burden of PLC.

cPLC was also confirmed to be an independent prognostic factor for five-year PFS. Pritanka et al. used focal, diffuse, and bilateral PLC to express extent or distribution of PLC^9^. Among them, focal PLC meant that the PLC was included in a lung lobe^3,9^. In this study, the three-grade system of cPLC was used instead of the four-grade system of cLy to represent the extent of PLC, but it has almost the same meaning as the focal, diffuse, and bilateral PLC in previous studies. In addition, it may be more useful to use the three-grade system of cPLC instead of the four-grade system of cLy because there were some cases where it was difficult to distinguish between cLy1 and cLy2 on CT images. Therefore, the three-grade system of cPLC is similar to that of the extent or distribution of PLC in some previous studies, and it is considered to be more useful because it is simpler than the four-grade system of cLy. In the present study, it was confirmed that prognosis for five-year PFS of cPLC3 was significantly worse than that of cPLC1 or cPLC2. It was found that the prognosis for PFS was significantly deteriorated in patients with PLC distribution throughout both lungs. Similarly, Im et al. confirmed that patients with PLC distribution throughout both lungs had a worse five-year OS than patients with intrapulmonary metastases in patients with NSCLC^2^. Therefore, cPLC3 has the potential of M1 descriptor. On the contrary, cPLC1 and cPLC2 have the possibility of T descriptor, but the previous study reported that the five-year OS in patients with PLC in other ipsilateral lobes does not significantly different from patients with intrapulmonary metastases^2^. Therefore, patients with cPLC1 are likely to be T descriptor, but patients with cPLC2 are required to be identified through further studies.

Clinical stage (IV vs. II, III) was identified as an independent prognostic factor for five-year PFS in the present study. Stage classification provides information that can effectively communicate the anatomical extent of cancer, and it is used as a valuable tool in estimating prognosis^12^. Stage IV NSCLC includes patients with malignant pleural effusion, malignant pericardial effusion, and intrathoracic or extrathoracic metastatic lesions^13^, therefore, stage IV suggests greater tumor extent than other stage I, II, and III. The median OS of patients with stage IV NSCLC ranges between 7.0 and 12.2 months depending on histology type, treatments, and other associated factors^14^. Özgür et al. reported that median survival months of stage IIIA, IIIB, IIIC, and IV were 24.60, 16.27, 16.13, and 8.07 months, respectively^15^. In the present study, median PFS of stage IV and stage II, III were 5.0 and 14.0 months, and these results show similar trends to previous studies. Furthermore, all six patients with clinical stage IV were classified as cPLC3 in this study. Since patients with pulmonary metastasis, malignant pleural effusion, malignant pericardial effusion, and extrathoracic metastasis were excluded from this study, extents of PLC are believed to have played an important role in determining advanced clinical stage.

In the univariate analyses for five-year OS, age was identified as a prognostic factor. In the present study, older patients had significantly worse prognosis for OS than younger patients. The incidence of lung cancer is related to age, but there is still controversy over the relationship between survival and age^16^. Similar to the results of this study, Matthew et al. found that age < 50 years is an independent prognostic factor of improved cause-specific survival in patients with NSCLC^17^, and Sunny et al. reported that advancing age is a much stronger negative prognostic factor of treatment than comorbidity in older veterans with NSCLC^18^. In addition, previous studies reported that younger age was inversely related to high N stage and M stage in patients with NSCLC^16,19^, but younger patients had a better prognosis^16^. On the contrary, survival of younger patients with NSCLC is unpredictably poor compared with other age groups, suggesting more aggressive disease biology^20^. For reasons related to this, gene mutations for EGFR and anaplastic lymphoma kinase (ALK) were related to cancer diagnosis at a younger age, and younger age was related to an increased frequency of targetable genotypes^20^. Since the results of this study confirmed that older patients with PLC had worse prognosis than younger patients with PLC, this could be one piece of evidence for older NSCLC patients to have worse prognosis than younger NSCLC patients. In addition, age, sex, clinical stage, cell type, primary tumor size, and primary tumor SUV_max_ were known as prognostic factors in previous studies of NSCLC patients^21–24^, but only age was identified as a prognostic factor for OS in this study. This may result from a statistical type II error of the small number of subjects in this study.

This study had several limitations. First, it was a small-scale, retrospective, single-center study, and statistical power may be inadequate due to the limited number of patients. Therefore, a large-scale, multi-center study is needed to validate the results of this study. Second, some patients in this study were diagnosed with PLC radiologically instead of with pathological confirmation. Although histopathology performed on specimens serves as the gold standard for diagnosis of PLC^4^, in this study, only 25 patients (50%) were pathologically identified with PLC by surgery (Supplementary Table S2). If general condition of patients does not allow for bronchoscopy or surgery, diagnosis of PLC can be made clinically and radiologically^7^. Third, infection or inflammation could affect FDG uptake in the lung. However, to minimize this, we excluded patients with infection, interstitial pneumonias, pneumoconiosis, sarcoidosis, pulmonary fibrosis, radiation-induced lung diseases, and parenchymal lung diseases in this study.

In conclusion, FDG PET/CT was a useful imaging modality for evaluating tumor burden of PLC, and FDG PET/CT parameters were identified as independent prognostic factors for radiologically diagnosed PLC in patients with NSCLC. In the present study, metabolic PLC burden, cPLC, and clinical stage were identified as independent prognostic factors for PFS. Metabolic PLC burden is a variable that contains information on tumor metabolism and extent of PLC, and high metabolic PLC burden had a poor prognosis for PFS. cPLC is a variable representing location and extent of PLC, and it was confirmed that the prognosis of cPLC3 was significantly worse than that of cPLC1 or cPLC2. Older patients had significantly worse prognosis for OS than younger patients. In this study, a combination of anatomical and metabolic information about PLC obtained using FDG PET/CT provides insight into the overall tumor burden of PLC and is useful in predicting prognosis.

## Materials and methods

### Study population

We retrospectively reviewed the electronic medical records of NSCLC patients with radiologically diagnosed PLC who underwent FDG PET/CT in our medical center between 2000 and 2016. We used a PLC cohort of a previously published paper in our medical center^2^. Among patients who histopathologically confirmed NSCLC during the study period, 1,356 patients with PLC identified on chest CT but without pulmonary metastases were included in the study. Of these patients, we excluded the patients (I) with malignant pleural/pericardial effusion or extrathoracic metastasis (n = 945), (II) with incomplete staging workup (n = 295), (III) with concurrent T4 disease in the same lobe where PLC confined (n = 13), (IV) who didn't undergo FDG PET/CT (n = 23), (V) who have already started treatment prior to FDG PET/CT (n = 12), and (VI) who underwent FDG PET/CT in other medical centers (n = 18). In addition, patients with clinical evidence of infection, other malignant lesions, interstitial edema, interstitial pneumonias, pneumoconiosis, sarcoidosis, pulmonary fibrosis, radiation-induced lung diseases, and parenchymal lung diseases were also excluded. Finally, we enrolled 50 study patients in the present study.

The present study was approved by the institutional review board (IRB) of Samsung Medical Center (IRB registration number: 2022-06-033-001). The IRB of Samsung Medical Center approved a waiver of informed consent requirements due to the retrospective nature of this study. This study was performed according to the guidelines of the Declaration of Helsinki as revised in 2013 and its later amendments or comparable ethical standards.

### FDG PET/CT imaging protocol

All patients fasted for at least 6 h prior to FDG PET/CT. FDG was intravenously injected when the blood glucose level was less than 200 mg/dL. The injection dose of FDG was set at 5.0 MBq/kg (135.14 μCi/kg), and FDG PET/CT scans were performed at 60 min after FDG injection. No intravenous or oral contrast agent was used during FDG PET/CT scans. In the present study, we performed FDG PET/CT scans using two different PET/CT scanners (Discovery LS and STE, GE Healthcare, Milwaukee, WI, USA). Of the 50 patients, 12 (24%) were examined using a Discovery LS PET/CT scanner and 38 (76%) were examined using a Discovery STE PET/CT scanner. The CT and PET images were obtained from the basal skull to the thigh. In the Discovery LS PET/CT scanner, acquisition parameters of CT images were a section width of 5 mm and 140 keV, 40–120 mAs adjusted to the patients’ weight. Whole-body CT was performed using an 8-slice helical CT and a continuous spiral method. Following the CT scans, emission scans were obtained for 4 min/frame. Attenuation-corrected PET images (Voxel size, 4.3 × 4.3 × 3.9 mm) were reconstructed using whole-body CT images and a two-dimensional ordered subsets expectation maximization algorithm with two iterations and 28 subsets. In the Discovery STE PET/CT scanner, acquisition parameters of CT images were a section width of 3.75 mm and 140 keV, 30–170 mAs with AutomA mode. Whole-body CT was performed using a 16-slice helical CT and a continuous spiral method. After the CT scans, emission scans were obtained for 2.5 min/frame. Attenuation-corrected PET images (Voxel size, 3.9 × 3.9 × 3.3 mm) were reconstructed using whole-body CT images and a three-dimensional ordered subsets expectation maximization algorithm with two iterations and 20 subsets. Standardized uptake valued (SUVs) were calculated after correction for the patient weight and injected dose of FDG. Co-registration CT and PET images was performed using Advantage Workstation Volume Share 7 workstation (Version 4.7, GE Healthcare).

### Image analysis methods

For image analysis, HRCT and FDG PET/CT, which were performed within two weeks before the start of treatment of the study patient, were used. After confirming radiological features of PLC and lobes containing PLC described in previous radiology reports of HRCT, we confirmed that location and number of lobes containing PLC, which were observed on HRCT images, were observed on CT images of PET/CT as well. In this study, HRCT was used as an auxiliary imaging modality to identify lobes containing PLC and radiological features of PLC, and image analysis of imaging parameters was performed on FDG PET/CT images. Representative radiological features of PLC incorporate thickening of the interlobular septa and peribronchovascular and subpleural interstitium, presence of polygonal lines, and secondary pulmonary lobules with relative preservation of the parenchymal interstitium on CT images^2,11,25^. Two experienced nuclear medicine physicians (Y.J.P. and J.Y.C.) reviewed the extent and radiological features of PLC and primary tumor through FDG PET/CT images. SUV_max_ of PLC and primary tumor was measure using Advantage Workstation Volume Share 7 workstation. Using 1.45 cm^3^ sized-spherical volume-of-interest (VOI) on FDG PET images, PLC SUV_max_ was measured several times in PLC area where radiological features of PLC were observed on CT images of PET/CT. In addition, PLC SUV_max_ was measured not to include FDG uptake of primary tumor. Primary tumor SUV_max_ was also measure several times to the highest SUV_max_ using 6.94 cm^3^ sized-spherical VOI, and primary tumor size was measured on CT images of PET/CT. If there was a difference in SUV_max_ measured by two physicians, it was determined in consensus.

### Clinical variables and imaging parameters

In this study, eight clinical variables and five imaging parameters were used for survival analysis. Clinical variables included age, sex, ALK mutation and EGFR mutation of tumor, and treatment modality. Herein, the treatment modality included curative-intent treatment and palliative treatment. Smoking status was comprised never smoked, ex-smoker, and currently smoking, and primary tumor histology consisted of adenocarcinoma, squamous cell carcinoma, and other NSCLCs. Clinical stage was set as a stage grouping through AJCC 8th edition of NSCLC^26^.

**Figure 4:**
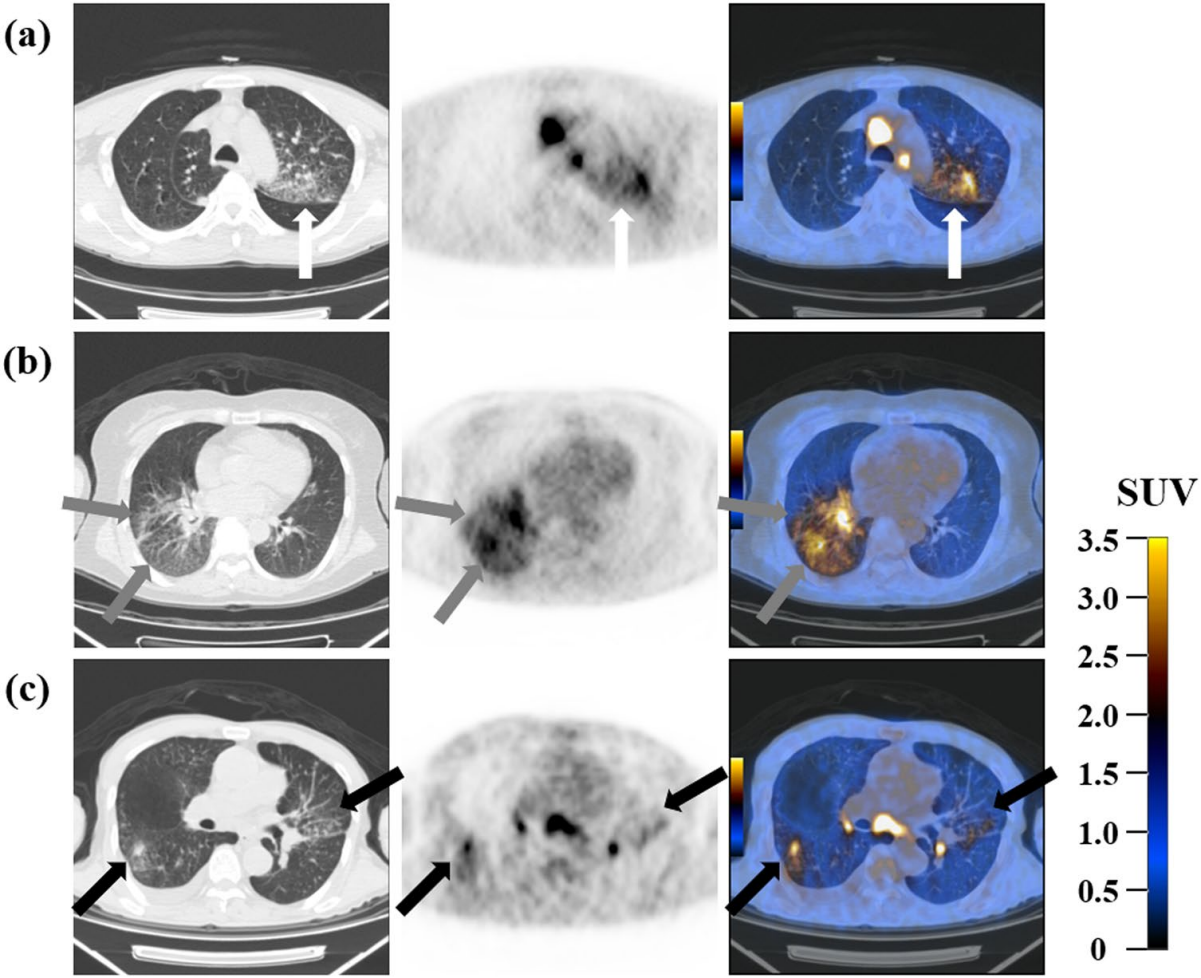
Representative FDG PET/CT images (CT, PET, and fusion images) of cPLC, classified as (**a**) cPLC1, (**b**) cPLC2, and (**c**) cPLC3. (**a**) The primary tumor was in left upper lobe and PLC was located only in the same lobe where the primary tumor is located (white arrows). PLC SUV_max_ in the left upper lobe and metabolic PLC burden were 3.27 and 3.27, respectively. (**b**) The primary tumor was in right lower lobe and PLC was confined to right lower lobe and right middle lobe (gray arrows). PLC SUV_max_ in right lower lobe and metabolic PLC burden were 3.06 and 6.12, respectively. (**c**) The primary tumor was in left upper lobe and PLC was distributed throughout both lungs (black arrows). PLC SUV_max_ in left lower lobe and metabolic PLC burden were 3.77 and 18.85, respectively. *FDG* fluorine-18-fluorodeoxyglucose, *PET* positron emission tomography, *CT* computed tomography, *PLC* pulmonary lymphangitic carcinomatosis, *SUV*_*max*_ maximum standardized uptake value.

Five imaging parameters included cPLC and number of lobes with PLC. Editors of IASLC Staging Project in 2015 proposed to classify lymphangitis carcinomatosis as an independent descriptor, cLy, with four-grades based on PLC extents^2,6,8^. Among them, cLy1 was defined as lymphangitis confined to the surrounding area of the primary tumor, and cLy2 was defined as lymphangitis at a distance from the primary tumor but confined to the same lobe of the primary tumor. In the present study, the extents of the PLC were simplified into a new three-grade system (cPLC) as follows: (I) cPLC1: lymphangitis confined to the lobe of the primary tumor; (II) cPLC2: lymphangitis in other ipsilateral lobes; (III) cPLC3: lymphangitis affecting the contralateral lung (Fig. 4). Among them, cPLC1 was used as a combination of cLy1 and cLy2, and cPLC2 and cPLC3 were used as the same definitions as cLy3 and cLy4. cPLC was a new optional descriptor for extents of PLC that was different from cLy, and c of cPLC meant a specific code for PLC as in cLy^6,10^. The specific code was proposed for prospective studies, and it was used to help register cases in a consistent way^10^. In addition, three PET parameters such as PLC SUV_max_, primary tumor SUV_max_, and metabolic PLC burden were used. Herein, metabolic PLC burden was defined as the product of PLC SUV_max_ and number of lobes with PLC in this study, and it can be thought of representing the overall metabolic burden of PLC.

### Statistical analysis

In the present study, continuous variables such as PLC SUV_max_, primary tumor SUV_max_, metabolic PLC burden, and age were converted into dichotomous variables using optimal cutoff values. The optimal cutoff values for continuous variables were determined using the MaxStat package (Maximally selected Rank Statistics, version 0.7–25, Torsten Hothorn, 2017) and R statistical software (version 4.1.3, R Core Team, 2022). MaxStat calculates the maximally selected log-rank statistic to determine the optimal cutoff values which provides the optimal separation into two grouping patients. Based on the optimal cutoff values, continuous variables were divided into the low and high score groups. In addition, the optimal cutoff values for continuous variables were determined for five-year PFS and OS, respectively. OS refers to the period of patient survival from the time of treatment initiation, and PFS refers to the time from treatment initiation until objective disease progression or death^27^. MedCalc statistical software (version 20.106, Ostend, Belgium) was used for five-year PFS and OS analyses. Univariate and multivariate analyses for PFS and OS were performed using Cox proportional-hazards regression with enter method. Variables with P values less than 0.05 in the univariate analyses were used for multivariate analyses. VIF quantifies degree of interrelationship of independent predictor for potentially robust contributions to multicollinearity in a multiple regression model^28^. VIF is one of variable selection methods, and it can minimize the multicollinearity among the variables. VIFs greater than 3.3 are suggested as an indication of multicollinearity, and also as an indication that a model is perhaps contaminated by common methods bias^29^. Therefore, if VIFs of the variables in multivariate analyses were greater than 3.3, new models were created consisting of variables with the confounding variable and the remaining VIFs less than 3.3. Multivariate analyses was performed when the VIFs of all variables in this newly created model were less than 3.3, and the newly created model can be considered free of common methods bias^29^. Finally, variables with a P value of 0.05 in multivariate analyses were considered independent prognostic factors for PFS or OS, and the variables were plotted using Kaplan–Meier methods with log-rank test. In addition, kappa coefficient was used to measure reproducibility of SUV_max_ parameters measured by two physicians. When kappa coefficient is 0.90 or higher, it means almost a perfect agreement^30^. All tests were two-sided, and P values less than 0.05 were considered statistically significant.

## Supplementary Information

Supplementary Information.

## Data Availability

The datasets generated and/or analyzed during the current study are not publicly available due to participant privacy concern but are available from the corresponding author on reasonable request.
