# Supplementary material for: Prognostic value of pretherapeutic FDG PET/CT in non-small cell lung cancer with pulmonary lymphangitic carcinomatosis
Source: Sci Rep. 2023 Jan 7;13:345. doi: 10.1038/s41598-022-24875-2 (PMC9825376; doi:10.1038/s41598-022-24875-2)
Supplement: Supplementary file 1 — Supplementary Information. [file 41598_2022_24875_MOESM1_ESM.docx]

<Original Research Article>

**Prognostic value of pretherapeutic FDG PET/CT in non-small cell lung cancer with pulmonary lymphangitic carcinomatosis**

Yong-Jin Park^1,2,7^, Yunjoo Im^3,7^, O. Jung Kwon^3^, Joungho Han^4^, Myung-Ju Ahn^5^, Jhingook Kim^6^, Sang-Won Um^3,7*^, and Joon Young Choi^1,7*^

^1^Department of Nuclear Medicine, Samsung Medical Center, Sungkyunkwan University School of Medicine, Seoul, Republic of Korea

^2^Department of Nuclear Medicine, Ajou University School of Medicine, Suwon, Republic of Korea

^3^Division of Pulmonary and Critical Care Medicine, Department of Medicine, Samsung Medical Center, Sungkyunkwan University School of Medicine, Seoul, Republic of Korea

^4^Department of Pathology, Samsung Medical Center, Sungkyunkwan University School of Medicine, Seoul, Republic of Korea

^5^Division of Hematology-Oncology, Department of Medicine, Samsung Medical Center, Sungkyunkwan University School of Medicine, Seoul, Republic of Korea

^6^Department of Thoracic and Cardiovascular Surgery, Samsung Medical Center, Sungkyunkwan University School of Medicine, Seoul, Republic of Korea

^7^These authors contributed equally: Yong-Jin Park, Yunjoo Im, Sang-Won Um, and Joon Young Choi

**Corresponding Author:** Joon Young Choi, MD, PhD

Department of Nuclear Medicine, Samsung Medical Center, Sungkyunkwan University School of Medicine, 81, Irwon-ro, Gangnam-gu, Seoul, Republic of Korea

**Telephone:** +82-2-3410-2648, **Fax:** + 82-2-3410-2639, **E-mail:** jynm.choi@samsung.com

**Co-corresponding Author:** Sang-Won Um, MD, MPH, PhD

Division of Pulmonary and Critical Care Medicine, Department of Medicine, Samsung Medical Center, Sungkyunkwan University School of Medicine, Seoul, Republic of Korea

**Telephone:** +82-2-3410-3429, **Fax:** + 82-2-3412-3996, **E-mail:** sangwonum@skku.edu

**Supplementary Table 1.** VIFs in multivariate analysis for PFS.

|  | VIF | | | |
| --- | --- | --- | --- | --- |
|  | Multivariate analysis | Model 1 | Model 2 | Model 3 |
| Treatment modality (Palliative treatment vs. Curative-intent treatment) | 2.009 | 1.292 | 1.768 | 1.308 |
| Primary tumor SUV_max_  (> 6.86 vs. ≤ 6.86) | 1.232 | 1.135 | 1.166 | 1.172 |
| Metabolic PLC burden  (> 8.39 vs. ≤ 8.39) | 5.670* | 1.187 |  |  |
| cPLC | 6.375* |  | 1.777 |  |
| Clinical stage  (IV vs. II, III) | 9.363* |  |  | 1.278 |

Abbreviations: VIF, variance inflation factor; PFS, progression-free survival; SUV_max_, maximum standardized uptake value; PLC, pulmonary lymphangitic carcinomatosis.

*VIF > 3.3.

**Supplementary Table 2.** Summary of diagnostic methods for PLC.

| Diagnostic methods |  | Number of patients (%) |
| --- | --- | --- |
| Radiologic diagnosis |  | 50 (100) |
| Pathological diagnosis | Surgery | 25 (50) |

Abbreviations: PLC, pulmonary lymphangitic carcinomatosis.
